# Supplementary material for: Vibrio Species in an Urban Tropical Estuary: Antimicrobial Susceptibility, Interaction with Environmental Parameters, and Possible Public Health Outcomes
Source: Microorganisms. 2021 May 7;9(5):1007. doi: 10.3390/microorganisms9051007 (PMC8151235; doi:10.3390/microorganisms9051007)
Supplement: Supplementary file 1 [file microorganisms-09-01007-s001.zip › Tables S1, S2, S3.pdf]

SUPPLEMENTARY MATERIAL

Vibrio species in an urban tropical estuary: antimicrobial susceptibility, interaction with environmental parameters and possible public health outcomes

Anna L. B. Canellas[1], Isabelle R. Lopes[1], Marianne P. Mello[2], Rodolfo Paranhos[2], Bruno F. R. de Oliveira[1], Marinella S. Laport*[1]

[1]Instituto de Microbiologia Paulo de Góes, Universidade Federal do Rio de Janeiro, Av. Carlos Chagas Filho, 373, Cidade Universitária, 21941-590. Rio de Janeiro, Brazil.

[2]Departamento de Biologia Marinha, Instituto de Biologia, Universidade Federal do Rio de Janeiro, Av. Prof. Rodolfo Rocco, 211, Cidade Universitária, 21941-617. Rio de Janeiro, Brazil.

* Corresponding author: marinella@micro.ufrj.br (M.S. Laport*); Phone: 55-21-2560-8344 / 133; Fax: 55-21-2560-8028.

1. SUPPLEMENTARY TABLES…..2

1. **Table S1**………………………………………………………………………………..2
2. **Table S2**……………………………………………………………………………..…3
3. **Table S3**……………………………………………………………………………......4

2. REFERENCES……………………………………………………………………………………..5

1. SUPPLEMENTARY TABLES

Table S1. Primers and cycling conditions used in this study for the detection of beta-lactam resistance genes

Gene	Primer	Sequence	Cycling conditions	Amplicon size (bp)	References
bla$_{GES}$	mGESF	5′-AGCAGCTCAGATCGGTGTTG-3′	95ºC/10′ + 30 cycles of 95ºC/30″, 54ºC/30″, 72ºC/45″ and final extension of 72ºC/10′	750	[1], [2]
	mGESR	5′-CCGTGCTCAGGATGAGTTG-3′			
bla$_{SHV}$	mSHVF	5′-CTTGACCGCTGGGAAACGG-3′		200	
	mSHVR	5′-AGCACGGAGCGGATCAACGG-3′			
bla$_{TEM}$	mTEMF	5′-CCCTTATTCCCTTTYTTGCGG-3′		650	
	mTEMR	5′-AACCAGCCAGCCWGAAGG-3′			
bla$_{CTX-M-1,2}$	mCTX-1,2F	5′-ATGTGCAGYACCAGTAA-3′		512	
	mCTX-1,2R	5′-CGCTGCCGGTTTTATCSCCC-3′			
bla$_{CTX-M-8}$	mCTX-8F	5′-AACRCRCAGACGCTCTAC-3′		333	
	mCTX-8R	5′-TCGAGCCGGAASGTGTYAT-3′			
bla$_{CTX-M-14}$	mCTX-14F	5′-GGTGACAAAGAGARTGCAACGGAT-3′		876	
	mCTX-14R	5′-TTACAGCCCTTCGGCGATGA-3′			

Table S2. Primers and cycling conditions used in this study for the detection of heavy metal resistance genes

Gene	Primer	Sequence	Cycling conditions	Amplicon size (bp)	References
merA	merA-F	5′-GTGCCGTCCAAGATCATGAT-3′	94ºC/4′ + 30 cycles of 94ºC/1′, 57ºC/1′, 72ºC/1′ and final extension of 72ºC/8′	933	[3]
	merA-R	5′-TAGCCYACRGTSGCSACYTG-3′			
	A1F	5′-ACCATCGGCGGCACCTGCGT-3′	95ºC/5′ + 35 cycles of 95ºC/1′, 63ºC/1′, 72ºC/1′ and final extension of 72ºC/10′	1226	[4]
	A5R	5′-ACCATCGTCAGGTAGGGGACCAA-3′			
cusB	czcB-I	5′-ATGAAAAAAATCGCGCTT-3′	94ºC/5′ + 35 cycles of 94ºC/30″, 47ºC/45″, 72ºC/1′ and final extension of 72ºC/10′	1200	[5]
	czcB-II	5′- ATGCGCATGGGTAGCACT-3′			
copA	cad1	5′-CAAAYTGYGCRGGHAARTTYGA-3′	94ºC/5′ + 30 cycles of 94ºC/1′, 47ºC/45″, 72ºC/1′ and final extension of 72ºC/5′	1058	[6]
	cad2	5′-AACTAATGCACAAGGACA-3′			
pbrA	pbrA-N	5′-ATGAGCGAATGTGGCTCGAAG-3′	96ºC/5′ + 39 cycles of 96ºC/1′, 58ºC/1′, 72ºC/ and final extension of 72ºC/10′	2400	[7]
	pbrA-C	5′-TCATCGACGCAACAGCCTCAA-3′			

*H = A or C or T; Y = C or T; R = A or G; W = A or T.

Table S3. Primer and cycling conditions used in this study for the amplification of virulence genes in *V. parahaemolyticus* strains

**Gene**	**Primer**	**Sequence**	**Cycling conditions**	**Amplicon size (bp)**	**References**
tdh	*tdh*-F<br>*tdh*-R	5′-CCAAATACATTTTACTTGG-3′<br>5′-GGTACTAAATGGCTGACATC-3′	94ºC/5′ + 30 cycles of 94ºC/1′, 55ºC/45″, 72ºC/45″ and final extension of 72ºC/7′	251	[8]
trh	*trh*-F<br>*trh*-R	5′-GGCTCAAAATGGTTAAGCG-3′<br>5′-CATTTCCGCTCTCATA TGC-3′	94ºC/5′ + 35 cycles of 94ºC/1′, 55ºC/1′, 72ºC/1′ and final extension of 72ºC/7′	250	
toxR	*toxR*-F<br>*toxR*-R	5′-GTCTTCTGACGCAATCGTTG-3′<br>5′-ATACGAGTGGTTGCTGTCATG-3′	94ºC/5′ + 35 cycles of 94ºC/1′, 63ºC/1′, 72ºC/30″ and final extension of 72ºC/7′	368	[9]

2. REFERENCES

1. Campana, E.H.; Xavier, D.E.; Petrolini, F.V.; Cordeiro-Moura, J.R.; Araujo, M.R.; Gales, A.C. Carbapenem-resistant and cephalosporin-susceptible: a worrisome phenotype among *Pseudomonas aeruginosa* clinical isolates in Brazil. *Braz. J. Infect. Dis*. **2017**, *21*(1), 57-62. https://doi.org/10.1016/j.bjid.2016.10.008
2. da Costa, W.F.; Giambiagi-de Marval, M.; Laport, M.S. *Shewanella* harboring antimicrobial and copper resistance genes in sea urchins (*Paracentrotus lividus*) from the Crozon peninsula (Brittany, France). *Infect. Genet. Evol*. **2020**, *85*, 104437. https://doi.org/10.1016/j.meegid.2020.104437
3. Deredjian, A.; Colinon, C.; Brothier, E.; Favre-Bonté, S.; Cournoyer, B.; Nazaret, S. Antibiotic and metal resistance among hospital and outdoor strain of *Pseudomonas aeruginosa*. *Res. Microbiol*. **2011**, *162*, 689-700. https://doi.org/10.1016/j.resmic.2011.06.007
4. De, J.; Ramaiah N.; Vardanyan, L. Detoxification of toxic heavy metals by marine bacteria highly resistant to mercury. *Mar. Biotechnol*. **2008**, *10*, 471-477. https://doi.org/10.1007/s10126-008-9083-z
5. Khan, Z.; Nisar, M.A.; Hussain, S.Z.; Arshad, M.N.; Rehman, A. Cadmium resistance mechanism in *Escherichia coli* P4 and its potential use to bioremediate environmental cadmium. *Appl. Microbiol. Biotechnol*. **2015**, *99*, 10745-10757. https://doi.org/10.1007/s00253-015-6901-x
6. Oger, C.; Bertheb, T.; Quilleta, L.; Barraya, S.; Chiffoleauc, J.F.; Petit, F. Estimation of the abundance of the cadmium resistance gene *cadA* in microbial communities in polluted estuary water. *Res. Microbiol.* **2001**, *152*, 671-678. https://doi.org/10.1016/S0923-2508(01)01246-3
7. Borremans, B.; Hobman, J.L.; Provoost, A.; Brown, N.L.; van-Der-Lelie, D. Cloning and functional analysis of the *pbr* lead resistance determinant of *Ralstonia metallidurans* CH34. *J. Bacteriol.* **2001**, *183*, 5651-5658. https://doi.org/10.1128/JB.183.19.5651-5658.2001
8. Tada, J.; Ohashi, T.; Nishimura, N.; Shirasaki, Y.; Ozaki, H.; Fukushima, S.; Takano, J.; Nishibuchi, M.; Takeda, Y. Detection of the thermostable direct hemolysin gene (*tdh*) and the thermostable direct hemolysin-related hemolysin gene (*trh*) of *Vibrio parahaemolyticus* by polymerase chain reaction. *Mol. Cell. Probes*. **1992**, *6*(6), 477-487. https://doi.org/10.1016/0890-8508(92)90044-X
9. Kim, Y.B.; Okuda, J.U.N.; Matsumoto, C.; Takahashi, N.; Hashimoto, S.; Nishibuchi, M. Identification of *Vibrio parahaemolyticus* strains at the species level by PCR targeted to the *toxR* gene. *J. Clin. Microbiol.* **1999**, *37*(4), 1173-1177.
