# Supplementary figures and images for: Vibrio Species in an Urban Tropical Estuary: Antimicrobial Susceptibility, Interaction with Environmental Parameters, and Possible Public Health Outcomes
Source: Microorganisms. 2021 May 7;9(5):1007. doi: 10.3390/microorganisms9051007 (PMC8151235; doi:10.3390/microorganisms9051007)

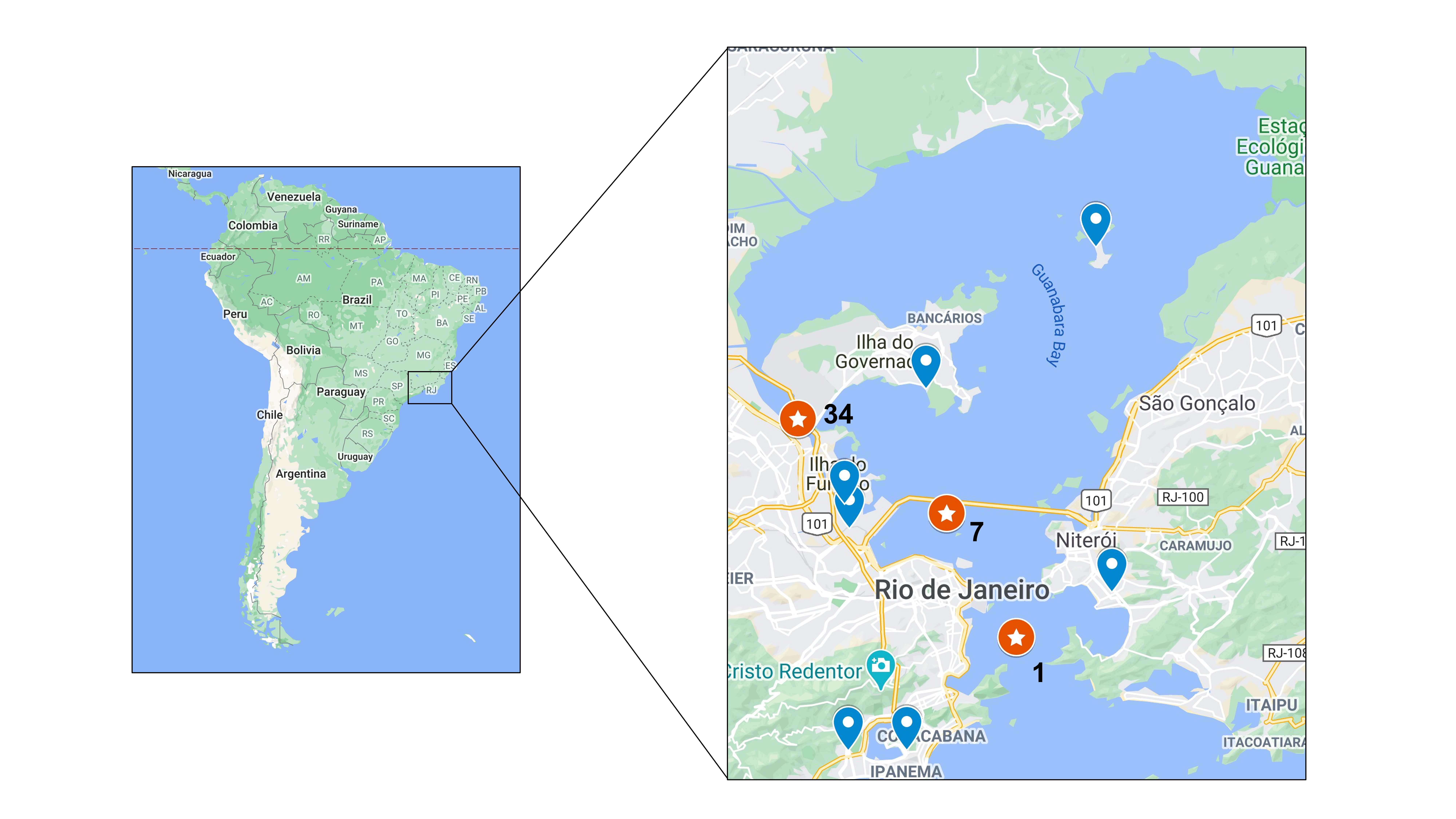

Supplement: Supplementary file 1 [file microorganisms-09-01007-s001.zip › Fig. S1.tif]

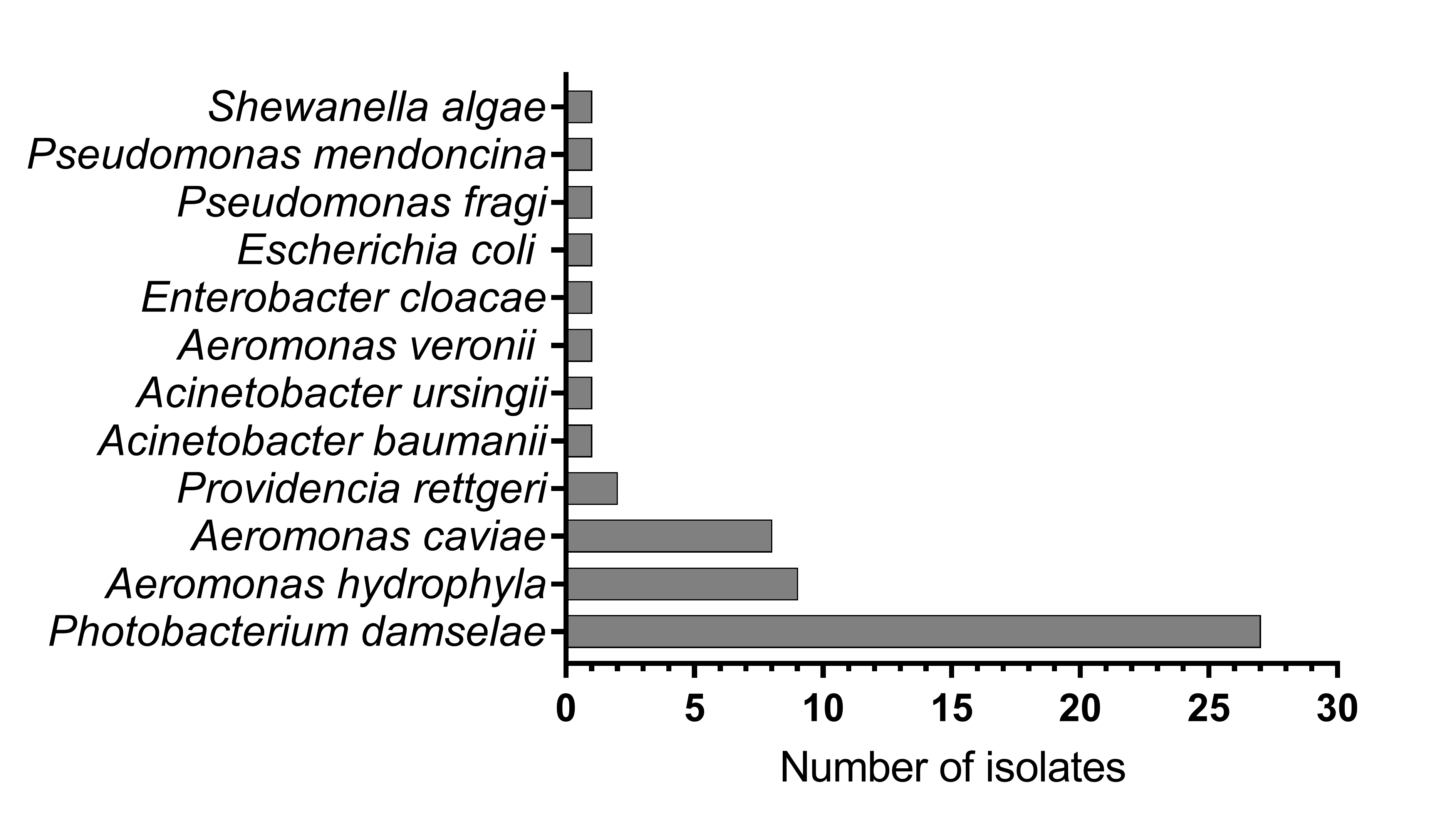

Supplement: Supplementary file 1 [file microorganisms-09-01007-s001.zip › Fig. S2.tif]
